# Hand Trauma and Reconstructive Microsurgery during the COVID-19 Emergency in the Marche Region (Italy): What Has Changed?

**DOI:** 10.3390/healthcare11233006

**Published:** 2023-11-21

**Authors:** Francesco De Francesco, Massimo Berdini, Pasquale Gravina, Pier Paolo Pangrazi, Giuseppe Signoriello, Michele Riccio

**Affiliations:** 1 Department of Reconstructive Surgery and Hand Surgery, AOU Ospedali Riuniti delle Marche, 60126 Ancona, Italy; pasquale.gravina@ospedaliriuniti.marche.it (P.G.); pierpaolo.pangrazi@ospedaliriuniti.marche.it (P.P.P.); michele.riccio@ospedaliriuniti.marche.it (M.R.); 2 Department of Clinical and Molecular Sciences, Clinical Orthopaedics, Polytechnic Marche University, 60126 Ancona, Italy; m.berdini@pm.univpm.it; 3 Department of Experimental Medicine, University of Campania “Luigi Vanvitelli”, 81100 Caserta, Italy; giuseppe.signoriello@unicampania.it

**Keywords:** COVID-19, hand trauma, hand surgery, replantation, SARS-CoV-2, pandemic

## Abstract

The novel severe acute respiratory syndrome coronavirus 2 (SARS-CoV-2), causing COVID-19, has spread across the globe. To limit the spread of COVID-19, the Italian government imposed various restrictions (lockdowns). These restrictions had an impact on the flow of patients accessing hospital care. Our aim in this study was to analyze the impact of lockdowns on the epidemiology of patients suffering from hand trauma. Our work analyzed the variation in the number and characteristics of hand trauma patients during the lockdown and half-lockdowns in 2020 compared to the same periods in the previous and subsequent years. In 2020, during the lockdown period, 107 patients were treated by our department for hand trauma, amounting to a 2% increase compared to the average number of patients treated in the pre-pandemic period. In 2020, during the half-lockdown period, 158 patients were treated, amounting to a 6.8% increase in comparison to the pre-pandemic period. During the lockdown period in Italy, the flow of patients suffering from hand trauma referred to our hub center remained stable. Given the restrictions imposed by the lockdown, we expected a consequent reduction in the number of work-related injuries, which did occur, while there was a surprising increase in the number of traffic-related injuries. The number of domestic accidents remained stable.

## 1. Introduction

The novel severe acute respiratory coronavirus 2 syndrome (SARS-CoV-2) causing coronavirus disease (COVID-19) has had an impact on medical practices worldwide, particularly those pertaining to infectious disease specialists [1]. Nevertheless, surgical activity for emergencies needed to be guaranteed. In this context, hand surgery and reconstructive microsurgery for trauma play a fundamental and unavoidable role [2]. On 11 March 2020, a pandemic state was declared by the World Health Organization (WHO) due to the spread of COVID-19, following the many cases recorded in 112 countries outside China, where the disease is presumed to have started, probably near the end of 2019 [3,4,5]. According to the latest data, in Europe, almost 2,245,500 people have died from COVID-19-related causes [6]. Italy was one of the first countries affected by the SARS-CoV-2 virus in terms of mortality. In Italy, the first traces of SARS-CoV-2 date back to 31 January 2020, when two foreign tourists tested positive for the virus in Rome. The spread of the virus was initially limited to northeastern Italy and then spread throughout the country, along with the first recorded deaths [7,8]. On 31 January 2020, the Italian government declared a state of emergency, and, with the decree issued on 9 March 2020, the restrictions become stricter, adopting measures such as the cessation of commercial activities, the limitation of social and aggregational activities, and the limitation of travel in order to stop the spread of infection; the lockdown phase had begun (the first wave). Despite its early adoption of the restrictions described, Italy was still one of the countries that was most affected by COVID-19 among those on the European continent. Due to the decreasing trend of the infection, the lockdown phase ended in May 2020. In September 2020, there was an increase in cases of contagion in Italy; for this reason, on 8 October, the Italian Ministry of Health officially declared the beginning of the second epidemic wave and indicated the adoption of restriction measures comparable to those of the first wave, namely, the lockdown phase, although lighter: the half-lockdown [9]. With the introduction of the vaccine, there was greater control of infection spread and a decrease in severe cases of COVID-19. The end of the state of emergency was declared on 1 April 2022. In the Marche region (Italy), on 30 July 2023, almost 719,400 people had been infected with the virus, and 4444 had died [10]. The Marche region was one of the most affected areas in terms of the number of people infected in Italy, and one of the first in terms of the diffusion of the infection among the four regions in the center of Italy [11]. The government directives had a great impact on the volume of workload in all Italian hospitals, and also in our region, in terms of the influx of traumatic patients. Planned surgical activities and non-urgent surgeries were canceled in many hospitals around the world for many specialistic surgeries (in our case, orthopedic and plastic procedures) to protect the health of both workers and patients, to spare hospital resources, and to use them for control and assistance with respect to COVID-19 cases. In many centers, especially those acting as hubs for specialized surgeries, the flow of traumatic injuries never stopped, despite the medical emergency and the related restrictions. Surgery for trauma involving the upper limb and requiring a high degree of specialization, including reconstructive microsurgery, is one of the surgical activities that was not suspended [12].

### Management of Patients

The main conditions of the hand and the upper and lower limbs that require immediate surgical care (within 3 h of the trauma) are severe complex trauma (more than three damaged tissues), infections, and limb amputations [13]. Isolated tendon, ligament, bone, and nerve traumas can be treated within 24 h of the trauma being inflicted [14,15]. In our protocols during the COVID-19 emergency, patients who were candidates for urgent surgery (within 3 h of trauma) underwent a pharyngeal swab for SARS-CoV-2 in the emergency room as part of the screening process and subsequent confirmation with qualitative real-time reverse-transcription polymerase chain reaction (RT-PCR). Patients scheduled for surgery within 24 h of suffering trauma only underwent RT-PCR examination. The pharyngeal swab performed with these procedures was necessary for primary screening in order to be able to subject a patient to surgery with the appropriate preventive measures for the operating theater staff and the spread of infection in case they tested positive for SARS-CoV-2 [16,17,18]. All these procedures, although intended to ensure a safe working environment and patient safety, weighed heavily on the hospital workflow, and, despite this, most patients received the necessary surgical treatment with quality standards comparable to those of the pre-COVID-19 period.

The purpose of our study was to analyze the load of patients and their characteristics presenting to the Reconstructive and Hand Surgery Department (Regional Hub) “delle Marche” of Ancona during the first wave (lockdown) of the COVID-19 pandemic and the subsequent second wave (half-lockdown) compared to each other and in the same period in previous years. As a second goal, we investigated the types of trauma and the changes that were observed in terms of anatomical location, type of trauma, severity, and origin in 2020 and in previous and subsequent years.

## 2. Materials and Methods

Our work is a narrative observational study of the impact of COVID-19 on the epidemiology of hand trauma and reconstructive surgery at a hub center in Italy over two periods in 2020 compared to previous and subsequent years. This study was conducted at “AOU delle Marche” of Ancona, a regional hospital that receives trauma cases from the entire Marche region and adjacent areas, such as parts of Abruzzo, Molise and Umbria (catchment area of around 3,000,000 people). The Reconstructive and Hand Department (hub specialized in polytraumas and replantation) activities were reviewed and compared between two periods, namely the full Italian lockdown (from 9 March 2020 to 3 May 2020, referred as the lockdown period) and the half-lockdown (from 20 September to December, referred as the half-lockdown period), and the same periods from the previous years (referred as the 2017/2022 periods). This hub includes the supply of acute and elective services for upper and lower limb trauma and microsurgical and soft tissue reconstruction.

The study population included all patients who were surgically treated at our hospital via the emergency department and/or required the visit of a hand/reconstructive surgeon. Inpatients admitted for elective surgery were excluded. Data collected on patients admitted to our hospital also included their COVID-19 diagnosis status. COVID-19 was diagnosed in patients with or without fever, dyspnea or other respiratory symptoms who had a positive SARS-CoV-2 reverse-transcription polymerase chain reaction test. Patients found positive for SARS-CoV-2 were deferred. All patients diagnosed with COVID-19 were excluded from this study. The total number of admissions in the two periods was assembled to calculate the rate of surgical treatments performed.

Patients with amputations or sub-amputations requiring critical revascularization and those with fractures of the metacarpals and phalanges were surgically treated within 3 h of the emergency room visit. All of these patients were considered as potentially infected with COVID-19, so the surgeons and nurses in the surgical theater adopted the recommended protections for SARS-CoV-2-positive patients.

Usually, this type of injury requires several surgical treatments, carried out in several steps, to perform all the appropriate treatments. The cases requiring more than one surgical treatment to achieve healing were excluded from this study, as a potential source of bias.

The quantitative variables were age and date of admission. The qualitative variables included group, gender, the area where the injury occurred, the traumatic mechanism, the diagnosis, and the relationship with the activity. We included both adult patients (>18 years old) and young patients (<18 years old). As the data varied widely, we classified areas, traumatic mechanisms, and diagnoses into subgroups. Area was classified into domestic, work, street (roads and sidewalks), and others.

### Statistical Analysis

Measurements were performed between the two periods, and data were evaluated according to the descriptive analysis. The statistical analysis threshold was set at *p*-values of <0.05, with a 95% confidence interval. The comparison between periods, for categorical variables, was performed with the chi-square test and Fisher’s exact test when appropriate. The Cochran–Mantel–Haenszel test was used to evaluate the difference in the time trend across the periods considered to assess the independence between the various periods examined, using Statistical Analysis Software (Stata SE ver. 16, StataCorp LLC, College Station, TX, USA). The graphs and tabs were created with Excel Ver 2205, build 15225.20288 (Microsoft, Redmond, Washington, DC, USA).

## 3. Results

A total of 1609 patients’ medical records were analyzed. Of those, 63 patients were excluded from our analysis due to incorrect codification in the database and lack of data. Eighteen patients were excluded because even though they were admitted at first as patients to be treated by our department, by their decision, they continued treatment at another center. A total of 1528 patients’ medical records remained valid for our analysis. Considering any medical reasons, during the lockdown period in 2020, 107 patients were admitted to our ward or treated by our unit due to hand trauma and injuries or replantation, recording an increase in comparison to 2017, a decrease in comparison to 2018 and a substantial stable trend in comparison to 2019 and 2021 (Table 1).

During the half-lockdown period of 2020, 158 patients were admitted to our ward or treated by our unit, recording an increase in comparison to 2017, an increase in comparison to 2018 and a decrease in comparison to 2019 (Table 1).

In summary, in the lockdown period in 2020, 107 patients attended the hospital for hand trauma, a slight increase of 2% compared with the average number (104) of patients attending the hospital for hand trauma in the pre-pandemic period (sum of 2017–2019). During the half-lockdown period in 2020, 158 patients attended the hospital for hand trauma, an 8.6% increase compared with the average number (144.3) of patients attending the hospital for hand trauma in the pre-pandemic period.

Patients’ average ages displayed a general decrease of 4 years, going from an average of 51.7 years (range 25–55) in 2018 and 47.7 in 2019 to an average of 47.3 years (range 25–55) during the lockdown period in 2020. The decrease in the average age of patients is statistically significant between 2018 and 2020 (*p*-value < 0.05). Meanwhile, during the half-lockdown period in 2020, the average age of patients decreased by 6 years, going from an average of 53.9 years (range 25–55) in 2018 and 50.8 in 2019 to an average of 47.4 years (range 25–55). The decrease in the average age of patients is statistically significant between 2018 and 2020 (*p*-value < 0.05). Gender did not significantly change.

The injury rate in children (<18 years) changed in the lockdown period, with an increase of 4.8% between the previous years (2017–2019) and 2020. The injury rate in the same age group in the half-lockdown period also changed, with an increase of 1.8% from the previous years (2017–2019), and showed an upward trend in subsequent years (*p*-value < 0.001).

Among the 19–55 age group, in the lockdown period, we observed a decrease in the injury rate of 4.7% from the previous years compared to 2020; for the semi-lockdown period, we observed an increase of 5.1% from the previous years compared to 2020 (*p*-value < 0.001).

Among the >55 age group, we observed a similar trend regarding injury rate, with a decrease of 0.1% from the previous years compared to 2020 in the lockdown period; for the semi-lockdown period, we observed a decrease of 6.9% from the previous years compared to 2020 (*p*-value < 0.05).

**Figure 1:**
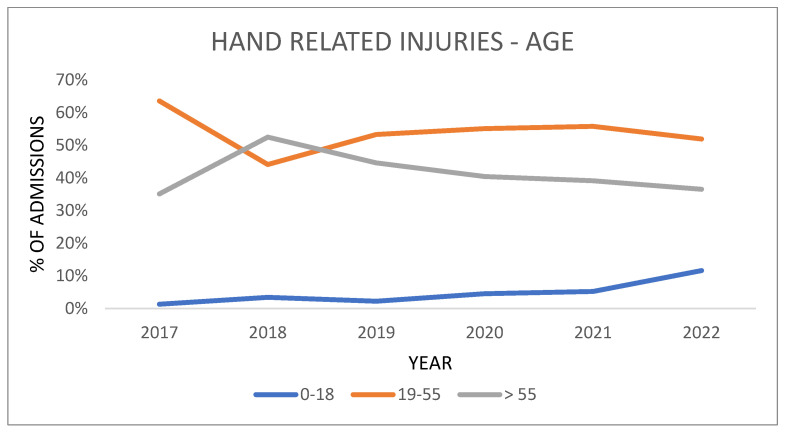
Percentage of admission of patients with hand-related injuries divided by age from 2017 to 2022. Blue line: 0–18 years; orange line: 19–55 years; gray line: >55 years.

In the graph, the merged data from the lockdown and semi-lockdown periods are analyzed and compared with previous and subsequent years. This analysis does not show the differences noted by stratifying patients for the two periods, but rather a more constant trend is observed (Figure 1).

Street/road accident traumas of the hand which were treated surgically registered the most significant increase during the two periods. Road accident traumas rapidly increased in prevalence in the lockdown period (+16%), and their proportion highly significantly increased from an average of 6.9% of all admissions in the pre-pandemic years (2017–2019) to an average of 24%. Also, in the half-lockdown period, we recorded a slight increase, although less than that described above, in hand trauma due to a road accident, going from an average of 6.9% in the pre-pandemic years to an average of 8.8% recorded in 2020 (*p* < 0.001).

Home hand trauma injuries recorded a stable average trend in the lockdown period, settling at 42.8% in the pre-pandemic years and 56.5% of all admissions in 2020. A similar trend to that observed for road trauma as a percentage was repeated in domestic trauma of the hand in the half-lockdown period, displaying a slight increase from an average of 42.8% in the pre-pandemic years to 49.1% in 2020 (*p* < 0.001).

**Figure 2:**
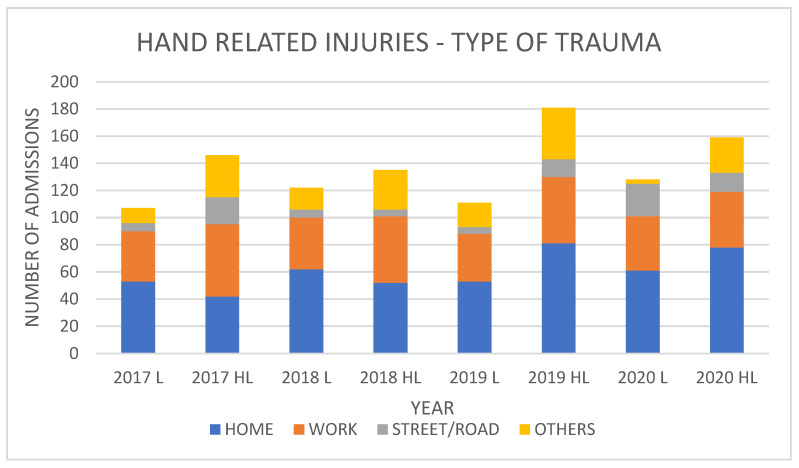
Patients with hand-related injuries divided into home, work, street, and other during the lockdown and half-lockdown periods (L: lockdown, HL: half-lockdown) from 2017 to 2020. Blue: injuries in the home environment; orange: injuries in the work environment; gray: street/road-related injuries; yellow: injuries in other environments (sport, public environment, other activities).

Injuries received at the workplace, residential/nursing homes, and shops, based on our observations, slightly dropped during the lockdown period, as well during the half-lockdown period: from 17.8% to 2.8% in the lockdown period, and from 17.8% to 16.6% in the half-lockdown period (*p* < 0.001) (Figure 2).

**Figure 3:**
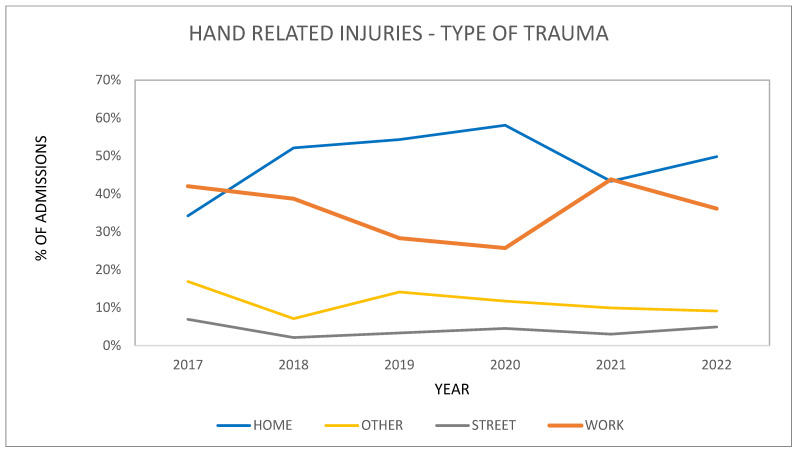
Patients with hand-related injuries divided into home, work, street, and other by admissions per year from 2017 to 2022. Blue: injuries in the home environment; orange: injuries in work environment; gray: street/road-related injuries; yellow: injuries in other environments (sport, public environment, other activities).

If we analyze the merged data from the lockdown and semi-lockdown periods compared with the previous and subsequent years, the differences noted by stratifying patients for the two periods do not become apparent, but rather a more constant trend is observed. Interestingly, when the data from the two periods are combined, a statistically significant decrease (*p* < 0.05) in home injuries and an increase in workplace injuries are observed in the years after 2020. These values tend to settle on the percentages recorded in the pre-pandemic era. The Cochran–Mantel–Haenszel test used to evaluate the trend between periods was statistically significant (*p* = 0.022) (Table 2, Figure 3).

Although occupational traumas decreased during lockdown, the rate of surgical treatments for digital amputations/sub-amputations remained stable.

**Figure 4:**
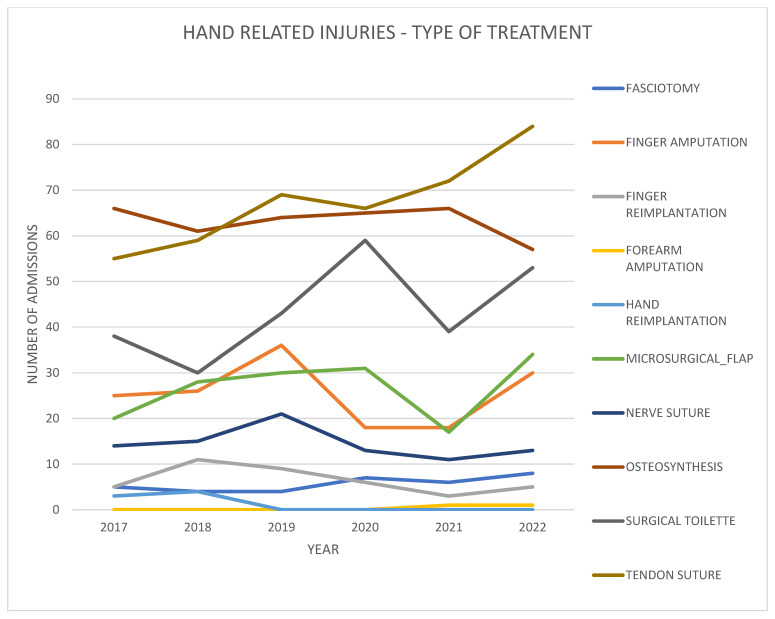
Patients with hand-related injuries divided into the type of trauma and subsequent surgical treatment performed per year from 2017 to 2022.

All others traumas, including open wounds with underlying non-critical injuries (nerve, tendon, bone), which by their nature did not require immediate treatment, did not register a significant variation in their rate during the lockdown period (*p* = 0.06) (Figure 4). In the two periods analyzed (lockdown, half-lockdown), no differences in operative waiting time for critical patients (amputations) were reported, while non-critical patients accumulated an operative waiting time of up to 24 h.

## 4. Discussion

The COVID-19 pandemic created a global emergency that triggered a state of alarm all over the world, and in Italy, it dramatically changed the national health, orthopedic and trauma service. To the best of our knowledge, the WHO did not give strict recommendations or guidelines for the set-up of surgical theaters or for the management of surgical treatments with respect to the COVID-19 pandemic. We adopted preventive measures, as indicated by a consensus of international hand surgeons [2], namely: (1) all non-emergency surgical procedures should be postponed to a later date (with certain result of molecular buffer); and (2) in the operating room, all members of the surgical team must conform to the same measures implemented by the infectious diseases department as established in our hospital. The COVID-19 pandemic inevitably changed the behavior of the whole population, confining people to the domestic environment, interrupting sports activities, and reducing road traffic and non-essential work activities, above all in the full lockdown period (in Italy, this was declared from 9 March to 3 May 2020). These restrictive strategies resulted in a gradual and consistent reduction in the number of new cases of infection due to SARS-CoV-2 and related hospital admissions for the advanced treatment of virus-related complications [19,20,21]. Nevertheless, the quarantine period had a strong psychological impact on the population. People were forced to make drastic changes to their daily lives and were at great risk of developing feelings of fear, discouragement, anxiety, and depression [22]. Quarantine was successfully implemented to stop the spread during the SARS outbreak in 2003 [23]. This was a significant step in this new pandemic situation. Quarantine can be imposed on a person or group and typically implements restrictions on residents or a specific area. During the quarantine period, all people carried out regular monitoring to verify the occurrence of any symptoms (i.e., fever, cough, headache, dyspnea) and communicated them to the local authorities identified by the authorities for the control of the spread of the virus. In cases where symptoms developed, the infected person had to be immediately isolated in a designated area with all essential treatment materials. Another element applied to prevent transmission was “social distancing”. This type of measure reduces interpersonal contact to a minimum within a larger population, where individuals may be infectious but have not yet been identified as infected persons and are thus not isolated. In this way, the social distancing of individuals effectively reduces the transmission of this type of infectious disease [24].

In our work, we describe the distribution of hand traumatic lesions, analyzing the database of University Hospital Company “AOU delle Marche” of Ancona (Marche Region, Italy), collecting the surgical procedures performed by the Reconstructive and Hand Surgery Unit during the lockdown and half-lockdown periods in 2020 and comparing these data to the same periods in 2017, 2018, 2019, 2021, and 2022. Our unit represents the hub center for replantation and microsurgical procedures of the hand and upper limb for the population living in the Marche region and neighboring regions, reaching a catchment area of about 3 million people. During the lockdown and half-lockdown periods in 2020, due to the government restrictions, travel outside the region or nation was not permitted, and for a long time, travel between or into adjacent municipalities was not permitted except for work or strictly necessary reasons (i.e., health emergency, urgent care) or to buy essential goods.

Despite the restrictions applied, our center had to face a considerable amount of work. Based on our data, we observed a stable, slightly increasing trend in domestic traumas. These data probably derive from the fact that many people spent their time performing home chores, cleaning, cooking, and bricolage, perhaps without skills or security measures; surprisingly, this does not seem to have given rise to an increased number of finger amputations or sub-amputations of the hand or fingers. In the literature, a variety of reports on domestic accidents during the COVID-19 pandemic have yielded different results. Blum et al. reported a decrease in all aspects of orthopedic and traumatological surgery. The overall number of patient visits and the number of emergency visits decreased by up to 90.1% and 74.2%, respectively [25]. Meanwhile, Giuntoli et al. [26] reported a 57.2% decrease in the number of domestic accidents, while the number of domestic hand and wrist injuries tripled according to Poggetti and colleagues [27]. This difference could be related to the fact that our study, due to the high specialization of our unit, reports data closely related to hand trauma and does not consider other orthopedic traumas.

Quarantine and travel restrictions were imposed by many countries around the world to slow the spread of infection and avoid the collapse of health systems [28]. Furthermore, the population was encouraged to stay at home. Consequently, the number of road/traffic accidents decreased in some countries; however, despite this, the data we collected showed an surprising increase in the number of street/road accident traumas related to the hand. Nunez et al. reported a 78.6% reduction in traffic accident admissions at a tertiary trauma center in Spain [29]. Other studies from Italy, the UK, India and the USA confirm a reduction in the number of traffic accidents with similar data [27,30,31,32,33,34]. A possible explanation for these data, apparently in contrast with the available literature, is that the road traumas occurring during the lockdown period were probably mainly borne by those people who were not subject to the suspension of work. Another important aspect is that our study does not consider other orthopedic traumas, only hand-related injuries.

An important aspect relating to both road-related and domestic trauma is that in the half-lockdown period, the data seem to return to “normal” levels, despite the milder restrictions; this trend is then confirmed by the data collected in the following years, where an actual return to levels comparable with those of the years before the COVID-19 occurs. To our knowledge, there are no data available in the literature about the period following the first lockdown to refer to.

In addition, admissions for sports injuries also declined by up to 100%, similarly to the data reported in the literature, because team sports were banned in many countries. From our database, we were not able to separate this type of data, labeled as “other”. Similar data were reported by Keays et al., but only in the pediatric population, and Staunton et al., who stratified the type of trauma causing entry into the emergency room during the COVID-19 pandemic [28,35].

An aspect to be considered when analyzing the reported data is closely linked to the territory where these data were collected. The Marche region has particular characteristics related to the geographical, demographic and orographic distribution of its territory, becoming a widespread urban territory intersecting the landscape and a rural culture still very rooted in the territory. This results in a large number of small, urbanized areas set within a purely rural area, and a few large cities to which most people flock due to the lack of public services [36,37]. This means that the culture of home improvement and gardening is still widespread, as many of the activities related to self-made agriculture are not carried out by professionals, but by the owners of the living spaces themselves. This could connect to the data we detected, which did not display any change in the prevalence of domestic traumas. Also, as the regional territory belongs to a single large metropolitan region, people often commute long distances for work, reaching over 100 km a day, without needing to move to larger and more populous cities. This aspect could explain how, according to the data we reported, during the lockdown period, not only was there a decrease in traumas related to vehicular traffic, but also an increase, being closely linked to travel for work and therefore not subject to restrictions.

It is also interesting to note that once the pandemic period had passed, when analyzing the aggregated data from the two periods, levels tended to return to pre-pandemic values.

Interesting data emerge from the stratification by age, where an inverted trend is observed between the two periods of lockdown and half-lockdown, especially for patients over the age of 18. In fact, in the lockdown period in 2020, there was a decrease for the 19–55 age group compared to the average for the previous years, and at the same time an increase, albeit only by 0.1%, for the age group of older patients. Similar data were reported in the work of Van Aert et al. [38] and Poggetti et al. [27]. In the half-lockdown period, there was an inverse trend, with an increase for patients aged between 19 and 55 years compared to the average for previous years, and a decrease of 6.8% for older patients regarding hospital admissions for hand trauma.

As regards the reported data on amputations and replantings, which maintained a normal trend, despite the lockdown period and the related restrictions, a possible explanation can be traced back to the territory of the Marche region. Considering the specificities of the Marche region’s production model, which is still very closely linked to the manufacturing sector, regional support policies seek to maintain the current levels of employment and manpower in the craft and small industry sector. The outbreak of the SARS-CoV-2 pandemic and the subsequent lockdown affected this economy and the artisans, and in order to save their commercial activities, they tried to keep production active even during the period of restriction declared by the government, while still respecting the current regulations [39,40,41]. The maintenance of such levels of work, including in the small manufacturing industry, could justify these data, although other works carry comparable data [42].

The present study has limitations, which include its single-center retrospective observation design. The data were collected based on the surgical treatments performed by our department, but we lack the relevant data about all procedures carried out in consultation when the patient then continued their hospitalization in other departments, as well as data on the general flow of patients into our hospital. Furthermore, the catchment area may have undergone substantial changes due to the pandemic, which we cannot describe accurately. The strengths of this study are that we analyzed data from three years besides 2020 and from two different periods in 2020 (lockdown, half-lockdown). We cannot accurately describe the long-term impact of COVID-19 and its related restrictions on trauma patient flow and its influence on hand and limb trauma, but we can state that the workload and patient care standards were maintained at pre-pandemic levels, despite the enormous effort made by the national health system to deal with the emergency.

## 5. Conclusions

During the COVID-19 pandemic starting from 9 March 2020, the “staying home message” was repeated several times, and as a deterrent, the measures adopted by the Italian government to limit the spread of the pandemic were very stringent and prolonged, in line with those of other countries of the European Union, limiting vehicular traffic, people’s mobility and, partially, daily life compared to the pre-pandemic period. Despite these restrictions, as far as our experience is concerned, the number of severe traumatic injuries referred to our hub center displayed a slight increase in the period of the pandemic, but showed a substantially stable trend for domestic traumas, a decrease in traumas at work, and, surprisingly, an increase in road-related injuries in 2020 during the COVID-19 pandemic, especially during the first wave. In addition, the epidemiology of hand-related trauma seemed to return to pre-COVID-19 levels during the half-lockdown period and subsequent years.

## Figures and Tables

**Table 1:** Patients admitted to our unit in the lockdown and half-lockdown periods.

	2017	2018	2019	2020	2021	2022
**Lockdown**	96	113	103	107	104	135
**Half-lockdown**	135	125	173	158	129	150
**Total**	*231*	*238*	*276*	*265*	*233*	*285*

**Table 2:** Results of the statistical analysis sorted by age, type of trauma and treatment performed. The table shows the results of the Cochran–Mantel–Haenszel test to evaluate the global trend from 2017 to 2022 (* *p* < 0.05).

		Year						
	2017	2018	2019	2020	2021	2022	*p*-Value *	*p*-Value for Trend *
**Number of admissions**	N = 231	N = 238	N = 276	N = 265	N = 233	N = 285		
**Age**							<0.001	
0–18	3 (1.3%)	8 (3.4%)	6 (2.2%)	12 (4.5%)	12 (5.2%)	33 (11.6%)		
19–55	147 (63.6%)	105 (44.1%)	147 (53.3%)	146 (55.1%)	130 (55.8%)	148 (51.9%)		
>55	81 (35.1%)	125 (52.5%)	123 (44.6%)	107 (40.4%)	91 (39.1%)	104 (36.5%)		
**Type of Trauma**							<0.001	***p*** **= 0.022**
Domestic	79 (34.2%)	124 (52.1%)	150 (54.3%)	154 (58.1%)	101 (43.3%)	142 (49.8%)		
Other	39 (16.9%)	17 (7.1%)	39 (14.1%)	31 (11.7%)	23 (9.9%)	26 (9.1%)		
Street	16 (6.9%)	5 (2.1%)	9 (3.3%)	12 (4.5%)	7 (3.0%)	14 (4.9%)		
Work	97 (42.0%)	92 (38.7%)	78 (28.3%)	68 (25.7%)	102 (43.8%)	103 (36.1%)		
**Treatment**							0.058	***p*** **= 0.014**
Fasciotomy	5 (2.2%)	4 (1.7%)	4 (1.4%)	7 (2.6%)	6 (2.6%)	8 (2.8%)		
Finger amputation	25 (10.8%)	26 (10.9%)	36 (13.0%)	18 (6.8%)	18 (7.7%)	30 (10.5%)		
Finger reimplantation	5 (2.2%)	11 (4.6%)	9 (3.3%)	6 (2.3%)	3 (1.3%)	5 (1.8%)		
Forearm amputation	0 (0.0%)	0 (0.0%)	0 (0.0%)	0 (0.0%)	1 (0.4%)	1 (0.4%)		
Hand reimplantation	3 (1.3%)	4 (1.7%)	0 (0.0%)	0 (0.0%)	0 (0.0%)	0 (0.0%)		
Microsurgical flap	20 (8.7%)	28 (11.8%)	30 (10.9%)	31 (11.7%)	17 (7.3%)	34 (11.9%)		
Nerve suture	14 (6.1%)	15 (6.3%)	21 (7.6%)	13 (4.9%)	11 (4.7%)	13 (4.6%)		
Osteosynthesis	66 (28.6%)	61 (25.6%)	64 (23.2%)	65 (24.5%)	66 (28.3%)	57 (20.0%)		
Surgical toilette	38 (16.5%)	30 (12.6%)	43 (15.6%)	59 (22.3%)	39 (16.7%)	53 (18.6%)		
Tendon suture	55 (23.8%)	59 (24.8%)	69 (25.0%)	66 (24.9%)	72 (30.9%)	84 (29.5%)		

## Data Availability

Data are contained within the article.
